# Supplementary material for: Discordance, accuracy and reproducibility study of pathologists’ diagnosis of melanoma and melanocytic tumors
Source: Nat Commun. 2025 Jan 17;16:789. doi: 10.1038/s41467-025-56160-x (PMC11742048; doi:10.1038/s41467-025-56160-x)
Supplement: Supplementary file 1 — Supplementary Information [file 41467_2025_56160_MOESM1_ESM.pdf]

Supplementary Material

Supplementary Figure 1. Exemplary illustration of the CytoBrowser online environment. The environment was customized for our study and used for the expert-panel validated labeling.

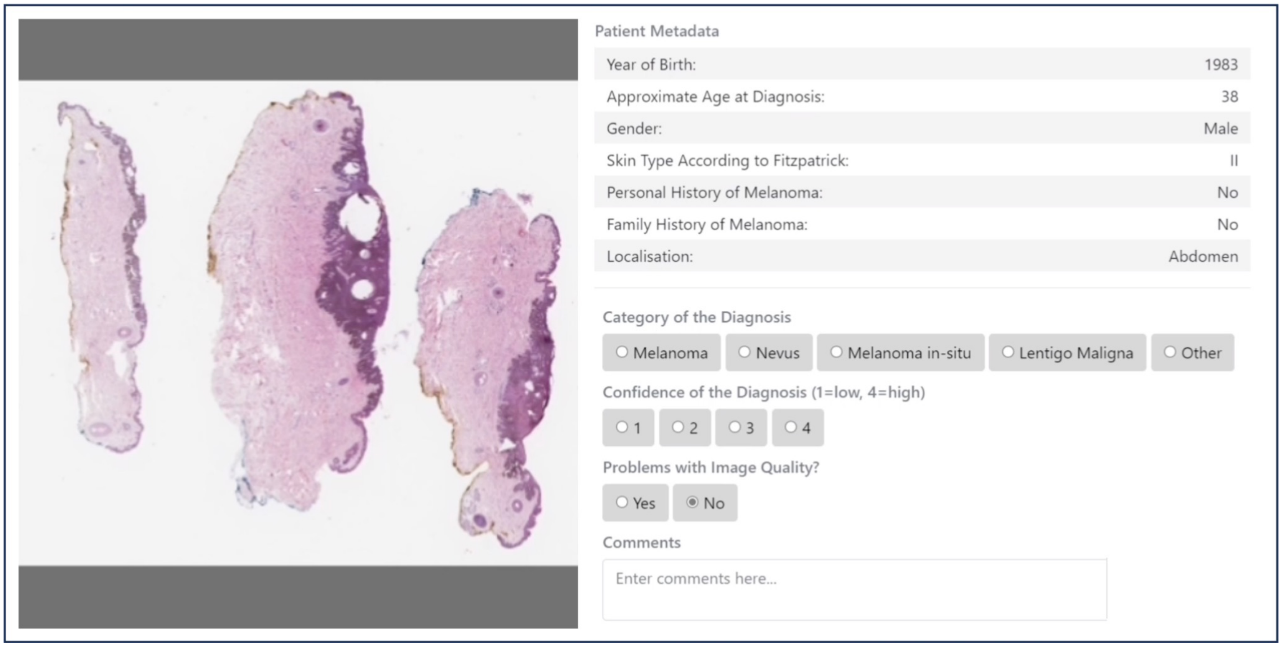

Supplementary Table 1. Subgroup analysis for quantifying interrater variability. Diagnostic categories are determined based on the corresponding expert panel-validated majority vote. Subgroups with fewer than 10 samples are excluded due to limited representativeness. The exact sample size of each subgroup is reported in **Table 2**.

		Overall n=792	IM n=199	NIM n=73	Nevus n=373	Others n=147
Age at diagnosis						
	<35	0.655 (0.630-0.681)			0.684 (0.647-0.721)	
	35-54	0.659 (0.641-0.677)	0.695 (0.669-0.722)		0.678 (0.651-0.704)	0.802 (0.775-0.829)[a]
	55-74	0.647 (0.633-0.660)	0.696 (0.673-0.718)	0.382 (0.360-0.404)	0.674 (0.652-0.696)	0.781 (0.759-0.803)
	>74	0.695 (0.680-0.709)	0.742 (0.717-0.767)	0.478 (0.453-0.502)	0.655 (0.630-0.680)	0.821 (0.796-0.846)
Fitzpatrick skin type						
	I	0.716 (0.687-0.746)	0.767 (0.721-0.813)		0.773 (0.727-0.819)[a]	
	II	0.675 (0.664-0.686)	0.695 (0.678-0.713)	0.428 (0.411-0.446)	0.741 (0.724-0.759)	0.771 (0.753-0.788)
	III	0.722 (0.706-0.737)	0.753 (0.728-0.777)	0.450 (0.425-0.474)[a]	0.724 (0.699-0.748)	0.812 (0.787-0.836)
Lesion localization						
	Face/scalp/neck	0.682 (0.663-0.701)	0.583 (0.550-0.615)[a]	0.533 (0.501-0.565)	0.760 (0.728-0.792)[a]	0.812 (0.780-0.844)
	Palms/soles	0.750 (0.696-0.804)[a]			0.781(0.696-0.866)[a]	
	Upper extremities	0.675 (0.653-0.697)	0.759 (0.722-0.796)	0.444 (0.407-0.480)[a]	0.700 (0.664-0.737)	0.769 (0.733-0.806)[a]
	Lower extremities	0.773 (0.752-0.793)	0.820 (0.789-0.850)		0.813 (0.782-0.843)	0.814 (0.784-0.844)[a]
	Back	0.621 (0.606-0.637)	0.663 (0.639-0.688)	0.301 (0.276-0.325)[a]	0.648 (0.624-0.672)	0.789 (0.765-0.814)

	Abdomen	0.653 (0.621-0.685)	0.728 (0.682-0.775)[a]		0.674 (0.628-0.721)	
	Chest	0.740 (0.711-0.768)	0.802 (0.758-846)[a]		0.776 (0.732-0.820)	0.747 (0.703-0.791)[a]

[a] Results must be interpreted with caution due to a sample size of fewer than 25.

Supplementary Table 2. Results for κ based on the independent datasets. We randomly divided the 108 lesions from the 54 patients contributing with two lesions into two distinct subsets. We calculated κ and the corresponding 95% CIs twice: once ignoring the first subset of duplicated patient lesions and once ignoring the second subset.

Dataset	κ
Whole dataset (n=792)	0.701 (0.693-0.710)
Independent subset 1 (n=738)	0.691 (0.683-0.700)
Independent subset 2 (n=738)	0.700 (0.691-0.708)

Supplementary Table 3. Overview of histopathological diagnostic categories and included subdiagnoses.

Histopathological Diagnostic Categories	Included histopathological Subdiagnosis
Nevus	• Spitz nevi and variants<br>• Dysplastic nevi/Clark nevi<br>• Acral nevi with palmar-plantar localization<br>• Recurrent nevi<br>• Blue nevi<br>• Combined nevi<br>• Other types of nevi
Invasive melanoma	• Superficially spreading melanoma<br>• Nodular melanoma<br>• Lentigo maligna melanoma<br>• Acral lentiginous melanoma<br>• Desmoplastic/scar-like melanoma<br>• Spitzoid melanoma (melanoma with features of a Spitz nevus)<br>• Nevoid melanoma<br>• Combined/compound forms<br>• Other types of melanoma
Non-invasive melanoma	• Melanoma in-situ<br>• Lentigo maligna
Other diagnostic outcomes	• Actinic keratosis<br>• Squamous cell carcinoma/Spinalioma<br>• Basal cell carcinoma<br>• Benign keratoses (e.g., seborrheic keratosis, lichen-planus-like keratosis)<br>• Dermatofibroma<br>• Vascular lesions (e.g., hemangioma)<br>• Miscellaneous (e.g., Merkel cell carcinoma)
