# Supplementary material for: Discordance, accuracy and reproducibility study of pathologists’ diagnosis of melanoma and melanocytic tumors
Source: Nat Commun. 2025 Jan 17;16:789. doi: 10.1038/s41467-025-56160-x (PMC11742048; doi:10.1038/s41467-025-56160-x)
Supplement: Supplementary file 2 — Reporting Summary [file 41467_2025_56160_MOESM2_ESM.pdf]

nature portfolio

Corresponding author(s): PD Dr. med. Titus J. Brinker

Last updated by author(s): 29/12/24

Reporting Summary

Nature Portfolio wishes to improve the reproducibility of the work that we publish. This form provides structure for consistency and transparency in reporting. For further information on Nature Portfolio policies, see our Editorial Policies and the Editorial Policy Checklist.

Statistics

For all statistical analyses, confirm that the following items are present in the figure legend, table legend, main text, or Methods section.

n/a	Confirmed	
☐	☒	The exact sample size (*n*) for each experimental group/condition, given as a discrete number and unit of measurement
☒	☐	A statement on whether measurements were taken from distinct samples or whether the same sample was measured repeatedly
☐	☒	The statistical test(s) used AND whether they are one- or two-sided<br>*Only common tests should be described solely by name; describe more complex techniques in the Methods section.*
☐	☒	A description of all covariates tested
☒	☐	A description of any assumptions or corrections, such as tests of normality and adjustment for multiple comparisons
☐	☒	A full description of the statistical parameters including central tendency (e.g. means) or other basic estimates (e.g. regression coefficient) AND variation (e.g. standard deviation) or associated estimates of uncertainty (e.g. confidence intervals)
☒	☐	For null hypothesis testing, the test statistic (e.g. *F*, *t*, *r*) with confidence intervals, effect sizes, degrees of freedom and *P* value noted<br>*Give P values as exact values whenever suitable.*
☒	☐	For Bayesian analysis, information on the choice of priors and Markov chain Monte Carlo settings
☒	☐	For hierarchical and complex designs, identification of the appropriate level for tests and full reporting of outcomes
☒	☐	Estimates of effect sizes (e.g. Cohen's *d*, Pearson's *r*), indicating how they were calculated

Our web collection on statistics for biologists contains articles on many of the points above.

Software and code

Policy information about availability of computer code

Data collection	The digitized H&E reference slides of all prospectively and consecutively collected lesions were provided using CytoBrowser, an online platform customized for our study.
Data analysis	All statistical analyses were performed using SPSS version 29.0.0.0 (IBM Corporation) and R version 4.1.2.

For manuscripts utilizing custom algorithms or software that are central to the research but not yet described in published literature, software must be made available to editors and reviewers. We strongly encourage code deposition in a community repository (e.g. GitHub). See the Nature Portfolio guidelines for submitting code & software for further information.

Data

Policy information about availability of data

All manuscripts must include a data availability statement. This statement should provide the following information, where applicable:

- Accession codes, unique identifiers, or web links for publicly available datasets
- A description of any restrictions on data availability
- For clinical datasets or third party data, please ensure that the statement adheres to our policy

External research projects may request access to the prospectively and consecutively collected, panel-validated dataset utilized in our study, specifically for the purpose of advancing skin (cancer) research. Access is granted following an application and approval process managed by the SCP Data Protection Committee, which evaluates requests based on criteria such as alignment with patient consent, a valid ethics vote, and other relevant requirements (i.e., non-comercial (skin) cancer research). All remaining data is available in the article, supplementary and source data files. Commercial use of the dataset is strictly prohibited.

Research involving human participants, their data, or biological material

Policy information about studies with human participants or human data. See also policy information about sex, gender (identity/presentation), and sexual orientation and race, ethnicity and racism.

Reporting on sex and gender	The sex of the participating patients was collected (physician-reported).<br>The sex of the expert panel was documented (self-reported).
Reporting on race, ethnicity, or other socially relevant groupings	The skin type of the participating patients according to Fitzpatrick was collected (physician-reported).
Population characteristics	The inclusion criteria required patients to be at least 18 years old and present with pigmented melanoma-suspicious skin lesions, either as clinical primary diagnosis or as secondary/differential diagnosis with the intent of ruling out melanoma. The exclusion criteria prohibited the enrollment of pre-biopsied lesions or lesions located under the finger- or toenails. Additionally, patients with person-identifying features (e.g. tattoos) in the immediate vicinity of the lesions were excluded due to data privacy concerns.
Recruitment	Study participants were prospectively enrolled into the Skin Classification Project (SCP2) across eight university hospitals in Germany (Berlin, Dresden, Erlangen, Essen, Mannheim, Munich, Regensburg, Wuerzburg) from April 2021 to February 2023.
Ethics oversight	Ethics approval was obtained from the ethics committee at the Technical University of Dresden (BO-EK-53012021), the Friedrich-Alexander University Erlangen-Nuremberg (69_21 Bc), the LMU Munich (21-0182), the University of Regensburg (20-2190-103), the Julius-Maximilians University Wuerzburg (293/20_z) and from the University Hospitals Mannheim (2020-656N) and Essen (20-9784-BO). Patients provided informed written consent.

Note that full information on the approval of the study protocol must also be provided in the manuscript.

Field-specific reporting

Please select the one below that is the best fit for your research. If you are not sure, read the appropriate sections before making your selection.

☒ Life sciences ☐ Behavioural & social sciences ☐ Ecological, evolutionary & environmental sciences

For a reference copy of the document with all sections, see nature.com/documents/nr-reporting-summary-flat.pdf

Life sciences study design

All studies must disclose on these points even when the disclosure is negative.

Sample size	800 study participants were prospectively enrolled across eight university hospitals in Germany. Sample size planning was performed in cooperation with the Biostatistic department at the German Cancer Research Center (DKFZ) based on previous prospective data collections with the participating clinics.
Data exclusions	A total of 792 eligible slides from 736 patients were analyzed and are included in the provided dataset. Eight slides had to be excluded post-hoc due to inconsistencies in data collection or data quality.
Replication	Replication was not deemed necessary, as the prospective data was collected over an extended period across multiple hospitals of varying sizes and from different regions in Germany. This approach ensured that the dataset reflects real-life, representative conditions, providing robust and generalizable findings.
Randomization	Since all participating pathologists reviewed the complete sample, no randomisation was necessary.
Blinding	Since all participating pathologists reviewed the complete sample, no blinding was necessary.

Reporting for specific materials, systems and methods

We require information from authors about some types of materials, experimental systems and methods used in many studies. Here, indicate whether each material, system or method listed is relevant to your study. If you are not sure if a list item applies to your research, read the appropriate section before selecting a response.

Materials & experimental systems

n/a	Involved in the study
☒	☐ Antibodies
☒	☐ Eukaryotic cell lines
☒	☐ Palaeontology and archaeology
☒	☐ Animals and other organisms
☒	☐ Clinical data
☒	☐ Dual use research of concern
☒	☐ Plants

Methods

n/a	Involved in the study
☒	☐ ChIP-seq
☒	☐ Flow cytometry
☒	☐ MRI-based neuroimaging

Plants

Seed stocks	Not applicable.
Novel plant genotypes	Not applicable.
Authentication	Not applicable.
